# Atelocollagen promotes chondrogenic differentiation of human adipose-derived mesenchymal stem cells

**DOI:** 10.1038/s41598-020-67836-3

**Published:** 2020-06-30

**Authors:** Seon Ae Kim, Yoo Joon Sur, Mi-La Cho, Eun Jeong Go, Yun Hwan Kim, Asode Ananthram Shetty, Seok Jung Kim

**Affiliations:** 1 Department of Orthopedic Surgery, College of Medicine, The Catholic University of Korea, Seoul, Republic of Korea; 2 The Rheumatism Research Center, Catholic Research Institute of Medical Science, College of Medicine, The Catholic University of Korea, Seoul, Republic of Korea; 3 The Institute of Medical Sciences, Faculty of Health and Wellbeing, Canterbury Christ Church University, Kent, UK

**Keywords:** Preclinical research, Stem-cell research

## Abstract

Effective engineering approaches for cartilage regeneration involve a combination of cells and biomaterial scaffolds. Multipotent mesenchymal stem cells (MSCs) are important sources for cartilage regeneration. Atelocollagen provides a suitable substrate for MSC attachment and enhancing chondrogenic differentiation. Here, we assessed the chondrogenic potential of adipose tissue derived human MSCs (hMSCs) mixed with atelocollagen gel. We observed cell attachment, viability, and microstructures by electron microscopy over 21 days. The levels of Sox9, type II collagen, aggrecan, type I collagen, Runx2, type X collagen, ALP, Osterix, and MMP13 were measured by RT-qPCR. Cartilage matrix-related proteins were assessed by enzyme-linked immunosorbent assay (ELISA), histology, and immunohistochemistry. hMSCs of all groups exhibited well-maintained cell survival, distribution and morphology. Abundant type II collagen fibers developed on day 21; while *Sox9*, type II collagen, and aggrecan expression increased over time in the atelocollagen group. However, type I collagen, *RUNX2*, type X collagen (*CoL10A1*), *Osterix*, and ALP were not expressed. These results corroborated the protein expression detected by ELISA. Further, histological analysis revealed lacunae-like structures, while staining demonstrated glycosaminoglycan accumulation. Cumulatively, these results indicate that atelocollagen scaffolds improve hMSC chondrogenic differentiation and are a potential approach for cartilage regeneration.

## Introduction

Articular cartilage is hyaline cartilage with a thickness of 2–4 mm. It is mainly composed of chondrocytes and extracellular matrix (ECM), which is principally composed of water, collagen, proteoglycans, and other non-collagenous proteins. Collagen and proteoglycan can help to retain water within the ECM, which is critical to maintaining cartilage mechanical properties^1,2^. It is well known that the regenerative capacity of articular cartilage after damage or disease is limited by low chondrocyte density with an extremely slow metabolic rate, abundant ECM, and no vascularity^3^. Therefore, cartilage regeneration is one of the most critical challenges associated with regenerative medicine.

Many therapeutic approaches have been developed for cartilage regeneration, including osteochondral transfer^4^, bone marrow stimulation techniques such as microfracture, and cell-based therapies including autologous chondrocyte^5,6^ and mesenchymal stem cell (MSC) implantation. However, these approaches do not yield complete cartilage regeneration as newly formed tissues have fibro-cartilaginous tissue characteristics and are mechanically weak^7^. Therefore, cartilage regeneration strategies using appropriate cell sources and matrix enhancement should be developed.

Recently, cell-based tissue engineering techniques for cartilage regeneration using three-dimensional scaffolds have been reported^8–11^. Cell and scaffold therapies for cartilage regeneration are based on mixing cells such as chondrocytes, MSCs, or perichondrocytes with biomaterials such as alginate, fibrin, hyaluronic acid, and collagen membranes, among others. Furthermore, growth factors and cytokines such as TGF-β1, TGF-β2, TGF-β3, BMP-2, BMP-6, and BMP-7 can be used to enhance chondrogenic differentiation^12^.

MSCs for cartilage regeneration can be isolated from bone marrow, adipose tissue, muscle, synovium, and periosteum, as well as other sources^13^. Although bone marrow MSCs are commonly used for cartilage regeneration, harvesting bone marrow is a painful procedure. Besides, these cells have the propensity to form osseous tissue^14^. For these reasons, different types of MSCs derived from synovium, peripheral blood, adipose tissue, skin, or periosteum are used for cartilage regeneration^15–17^. Adipose-derived MSCs are considered suitable cells for cartilage regeneration as they have high chondrogenic potential. In addition, large numbers of fat cells can be obtained from liposuction or surgical procedures^18,19^.

For tissue regeneration, scaffolds can assist the delivery and localization of cells to the defected site. For cartilage regeneration, this scaffold should provide proper three-dimensional structures for implanted cell adhesion, proliferation, and ECM formation^20^. Natural biomaterials (agarose, alginate, chitosan, collagen, gelatin, fibrin, etc.) and synthetic biomaterials (poly-lactic-*co*-glycolic acids (PLGA), poly-caprolactone (PCL), polylactic acid, polyglycolic acid, polyethylene glycol, etc.) with diverse shapes (sponge, hydrogel, fibers, and micro-particles) are recommended as scaffolds for cartilage regeneration^11,21^.

PCL^22–24^ and PLGA^25,26^ are FDA approved for clinical use. Previous studies have reported that PLGA positively influences cell adhesion and proliferation, as well as increases survival rate and differentiation into cartilage in large defected areas^27–30^. However, these synthetic materials also induce an immune response and produce lactic acid following degradation, ultimately causing lactic-acid-induced inflammatory reactions^31^. Additionally, the use of biodegradable hyaluronic acid based scaffold for hyaline like cartilage regeneration in the knee is attracting significant attention^32^.

The development of surgical techniques using atelocollagen is progressing rapidly^33–35^, and excellent clinical results for cartilage repair using atelocollagen have been reported^36–38^.

Collagen is one of the major components of cartilage ECM, and collagen-based scaffolds can provide biocompatibility and a chondrogenic environment for functional cartilage regeneration. Thus, MSCs in collagen scaffolds could maintain a chondrocyte phenotype in chondrogenic culture conditions and produce new collagen^39–41^. As a specific type of collagen scaffold, atelocollagen can be obtained from collagen by removing telopeptides, which have antigenic activity. Therefore, it can be safely applied clinically for cartilage regeneration^42^. However, to date, basic studies to explain the effect of atelocollagen on cartilage regeneration are insufficient. Thus, the objective of this study was to determine whether human MSCs (hMSCs) mixed with an atelocollagen gel as a scaffold could exhibit enhanced chondrogenic potential for cartilage tissue engineering.

## Materials and methods

All experiments were performed in accordance with relevant guidelines and regulations.

### Scaffolds

Neutralized porcine type I atelocollagen gel (UBIOSIS, Seongnam, South Korea) at 3 concentration of 3 mg/ml was used as the scaffold (Suppl. Figure 1).

Type I atelocollagen, which was prepared by the salt precipitation method^43,44^, formed collagen fibers with a large diameter and low antigenicity. Of note, this approach does not apply heat and is, therefore, associated with a low risk of collagen denaturation. Additionally, type I atelocollagen is physiochemically and immunologically similar to collagen in vivo. As a result, the application of type I atelocollagen to patients has a low inflammatory reaction.

### Isolation of hMSCs

hMSCs from ten donors (mean age and standard deviation: 65 ± 5.31 years) were isolated from adipose tissues that were discarded as surgical waste during artificial joint replacement surgery. All experimental procedures were approved by the Institutional Review Board of Uijeongbu St. Mary's Hospital, The Catholic University of Korea (UC14CNSI0150). Informed consent was obtained from all participants. hMSCs were isolated according to a method described previously^45^. Briefly, immediately after harvesting, adipose tissue (3–5 mg) was transported to the laboratory in 50-mL falcon tubes (352070, BD, Franklin Lakes, NJ, USA) containing phosphate-buffered saline (PBS) (10010023, Gibco-Life Technologies, Carlsbad, CA, USA). Adipose tissue was washed with sterile PBS to remove contaminating debris and red blood cells. After removing PBS, the remaining tissue was added to a Petri dish. The adipose tissue was cut into small pieces using a sterile scalpel, and 0.1% collagenase (type I collagenase; Sigma-Aldrich Inc., St Louis, MO, USA) was added to the digestion solution in a Petri dish on a clean bench.

Small pieces of adipose tissue treated with 0.1% collagenase in 10 mL were transported to 50-mL falcon tubes and incubated at 37 °C for 2 h in a water bath with gentle agitation. Collagenase was inactivated with an equal volume of DMEM/10% fetal bovine serum (FBS; 10082147, Gibco-Life Technologies, Carlsbad, CA, USA). The mixture was then centrifuged at 600×*g* for 7 min. Fat tissue debris, oil, and supernatant (the upper layer) were discarded. The cellular pellet was resuspended in DMEM/10% FBS and filtered through a 100-μm mesh (352360, BD, Franklin Lakes, NJ, USA) filter to remove debris. Isolated hMSCs were centrifuged and resuspended in DMEM/10% FBS. The cell suspension was then seeded into a 75 T tissue culture flask (430641, Corning Costar, Inc., Corning, NY, USA) at a density of ∼1 × 10^6^ cells/cm^2^ and incubated in humidified air with 5% CO_2_ at 37 °C. After incubation, DMEM/10% FBS culture medium was replaced with fresh medium, and adherent cells were maintained for expansion.

### hMSC encapsulation in atelocollagen gel

After a confluent cell layer was formed (passage 3), hMSCs were detached using 0.25% (w/v) trypsin. hMSCs encapsulated with gel beads were produced at two different mixture ratios as follows: (1) 2 × 10^6^ hMSCs/0.8 mL, mixed with 0.2 mL thrombin in one syringe and 0.2 mL atelocollagen mixed with 0.8 mL fibrin in the other syringe, and (2) 2 × 10^6^ hMSCs/0.8 mL mixed with 0.2 mL thrombin in one syringe and with 1 mL fibrin in the other (Suppl. Figure 2). A Y-shaped catheter was connected to the two syringes for mixing. The mixture was added dropwise onto a Petri dish to form a bead shape with an average of 3.75 ± 0.209 × 10^4^ cells per bead (Suppl. Video 1). After 5 min, encapsulated hMSCs in gel beads were mechanically detached from the Petri dish and transferred into 6-well plates and incubated at 37 °C with 5% CO_2_ after the addition of chondrogenic differentiation medium and control medium (basal medium).

### Chondrogenic differentiation of beads in vitro

Encapsulated hMSCs in gel beads were divided into three groups according to the mixture composition and culture conditions as follows: (1) control I group (mixture of fibrin, hMSCs, and thrombin cultured in basal medium), (2) control II group (mixture of fibrin, hMSCs, and thrombin cultured in chondrogenic differentiation medium), and (3) atelocollagen group (mixture of fibrin, atelocollagen, hMSCs, and thrombin cultured in chondrogenic differentiation medium). Chondrogenic differentiation media consisted of Dulbecco’s modified Eagle’s medium–high glucose (DMEM-HG; 11965-084, Gibco-Life Technologies, Carlsbad, CA, USA) containing 10^−7^ M dexamethasone, 10 ng/mL transforming growth factor-beta 3 (TGF-β3), 100 µg/mL sodium pyruvate, 40 µg/mL proline, 25 µM ascorbic acid-2-phosphate, 100 U/mL penicillin, 100 µg/mL streptomycin, and 1% (v/v) ITS plus (5 µg/mL insulin, 5 µg/mL transferrin, 5 µg/mL selenous acid). All reagents except DMEM-HG were purchased from Sigma-Aldrich (St Louis, MO, USA). Culture media were changed every 2–3 days for 3 weeks.

### Cell viability and proliferation assessment

Cell viability was characterized using calcein acetoxymethyl ester (calcein-AM) and ethidium homodimer-1 (EthD-1) dyes (L3224, Thermo Fisher Scientific, Waltham, MA, USA) on days 0, 7, 14 and 21. Gel beads were washed with PBS, which was followed by the addition of 2 μM calcein acetoxymethyl ester and 4 μM ethidium homodimer. After 30 min of incubation in the dark, gel beads were washed with PBS before being observed under a fluorescence microscope (Olympus IX71, Tokyo, Japan).

To measure the cell proliferation, the hMSC beads from all groups were harvested on days 0, 7, 14, and 21 after chondrogenic differentiation culture conditions. The gel beads were washed twice with PBS and digested with 1 mg/mL type I collagenase solution for 3 h, then filtered through a 100-μm mesh filter to remove debris. Isolated cells were centrifuged and resuspended in PBS. The total cell numbers and viability of the cells at each time were measured.

### Measurements of bead size

Photographs of hMSCs gel beads were taken with a digital camera (Cannon, Tokyo, Japan) to measure gel bead sizes. Results were analyzed statistically using Excel® (Microsoft, USA).

### Microstructures of hMSCs encapsulated in atelocollagen gels

Microstructures of encapsulated hMSC gel beads were investigated using scanning electron microscopy (SEM) and transmission electron microscopy (TEM). Briefly, beads were fixed in Karnovsky fixative (2% glutaraldehyde, 2% paraformaldehyde) (Sigma-Aldrich Inc., St Louis, MO, USA) overnight and washed twice with 0.1 M phosphate buffer (for 30 min each).

#### SEM

Fixed samples were washed with 0.1 M phosphate buffer for 10 min and dehydrated in a gradient of low-density to high-density alcohol (50%, 60%, 70%, 80%, 90%, 95%, and 100%). After transition using isopentyl acetate, CPD (critical point dry: LEICA EM CPD300, Austria) was performed for 30 min to 1 h. Samples were observed using an FE-SEM (Merlin, Carl Zeiss, Germany) after coating them with an ion-coater (LEICA EM ACE 600, Austria).

#### TEM

Fixed samples were washed with 0.1 M phosphate buffer for 10 min and dehydrated in a graded series of alcohols from low- to high-density (50%, 60%, 70%, 80%, 90%, 95%, and 100%). Samples were incubated overnight at 4 °C in a series of graded EPON (Epoxy Embedding Medium kit, 45359, Sigma-Aldrich Inc., St Louis, MO, USA) and propylene oxide solutions at ratios of 1:4, 1:1, and 3:1. Next, samples were incubated with 100% EPON overnight at 4 °C.

Blocks were embedded in EPON, and thin sections of 200–250-nm were cut with an ultramicrotome. These slices were stained with 1% toluidine blue and re-trimmed for electron microscopic observation. Ultra-thin sectioned slices were placed on a copper grid, stained with uranyl acetate (6%) and lead citrate, and examined by TEM (transmission electron microscope: JEM-1011, JEOL) at 80 kV.

### Real time quantitative reverse transcriptase polymerase chain reaction (RT-qPCR)

hMSC beads from all groups were harvested on day 7, 14, and 21 after culture to determine the levels of chondrogenic differentiation genes: *Sox9* (SRY-BOX Transcription Factor 9), *aggrecan,* and *type II collagen* (*COL2A1*) for cartilage markers; *type I collagen* as a fibrocartilage marker, were analyzed.

hMSC beads from all groups were harvested on day 21 after culture; the following ossification markers were observed *type X collagen* (*CoL10A1*)*, RUNX2* (*runt-related transcription factor-2*), *ALP* (*alkaline phosphate*), *and Osterix.*

Total RNA was isolated from hMSC beads of all groups using the RNeasy mini kit (74104, Qiagen, Hilden, Germany). Isolated RNA (1 μg) was reverse transcribed into cDNAs using the cDNA Reverse Transcription Kit (205311, Qiagen, Hilden, Germany). Expression levels of mRNA were determined by RT-qPCR using glyceraldehyde-3-phosphate dehydrogenase (*GAPDH*) as a housekeeping gene. Primers employed for RT-qPCR are listed in Table 1. RT-qPCR reactions were performed using a GoTaq® qPCR Master mix (A6001, Promega, Madison, WI, USA) according to the manufacturer’s instructions. Average delta Ct values of triplicates were obtained, and the relative quantitation of gene expression was performed using the comparative 2^−ΔΔCt^ method.

**Table 1:** List of primers used in RT-qPCR for chondrogenic differentiation genes.

Gene (accession no.)	Primer sequence	Product size
Type II Collagen *(COL2A1)* (J00116.1)	5′-GTT CAC GTA CAC TGC CCT GA-3′ 5′-TGA CCC TCA AAC TCA TGC CTC-3′	162
Aggrecan (BC150624.1)	5′-AGT CAC ACC TGA GCA GCA TC-3′ 5′-TCT GCG TTT GTA GGT GGT GG-3′	482
Sox9 (NM000.46)	5′-AGG AAG TCG GTG AAG AAC GG-3′ 5′-AAG TCG ATA GGG GGC TGT CT-3′	275
Type I Collagen	5′-CCT CCT GGC TCT CCT GGT-3′	106
(NM_000088)	5′-AGG GAG ACC GTT GAG TCC AT-3′	
Type X Collagen	5′-GCT AAG GGT GAA AGG GGT TC-3′	107
(*COL10A1*) (NM_000493)	5′-CTC CAG GAT CAC CTT TTG GA-3′	
RUNX2	5′-CCG GTC TCC TTC CAG GAT-3′	122
(NM001278484.2)	5′-GGG AAC TGC TGT GGC TTC-3′	
Osterix (NM_152860)	5′-CCC AGG CAA CAC TCC TAC TC-3′	175
	5′-GGC TGG ATT AAG GGG AGC AAA-3′	
ALP (NM013059.1)	5′-CCT TGA AAA ATG CCC TGA AA-3′	191
	5′-CTT GGA GAG AGC CAC AAA GG-3′	
GAPDH (NM002046)	5′-TTG GTA TCG TGG AAG GAC TCA-3′ 5′-TGT CAT CAT ATT TGG CAG GTTT-3′	126

Sox9, SRY-BOX transcription factor 9; RUNX2, runt-related transcription factor-2; ALP, alkaline phosphatase.

### ELISA

After 21 days of differentiation, the supernatant of the culture medium was harvested after centrifugation to collect all proteins. Levels of proteins were analyzed: *aggrecan* and *type II collagen (COL2A1)* for cartilage markers; *type I collagen* a for fibrocartilage marker; MMP13 (Matrix metallopeptidase 13) for an ossification marker. The supernatant of media was measured using ELISA kits for aggrecan (LS-F5714, LSBio, Seattle, WA, USA), COL2A1 (type II collagen, LS-F26742, LSBio), type I collagen (LS-F26726, LSBio) and MMP13 (LS-F26160, LSBio) according to protocols provided by the manufacturer. Samples were read on an ELISA reader at a wavelength of 450 nm. All samples were analyzed in triplicate.

### Histological and immunohistochemical analysis of phenotype differentiation

All groups of encapsulated hMSCs in gel beads were fixed in 10% formalin (Sigma-Aldrich Inc, St. Louis, MO, USA) after being removed from the medium at 21 days of culture. After treatment with a graded series of alcohols (100%, 90%, 80%, 70%, and 60%), dehydrated and fixed beads were embedded in paraffin and embedded in paraffin blocks. These paraffin blocks were sliced to a thickness of 5 μm and stained with hematoxylin and eosin. Sections were also stained with Alcian blue pH 2.5 and toluidine blue to assess proteoglycan synthesis, as an indicator of cartilage matrix components.

Immunohistochemical analysis of type II and type I collagen was performed using the VECTASTAIN® ABC Kit (PK-6200, Vector Laboratories, CA, USA), rabbit polyclonal anti-collagen type II antibody (ab34712, Abcam, Cambridge, MA, USA), and rabbit monoclonal anti-collagen type I antibody (ab138492, Abcam, Cambridge, MA, USA). Then 5-μm slices of tissue were deparaffinized using xylene and alcohol. Tissue slices were pretreated with 1 mg/mL of pronase (10 1059 21 001, Roche, Basel, Kanton, Switzerland) for 1 h at 37 °C for epitope unmasking. To determine endogenous peroxidase activity, tissue slices were incubated in 0.3% H_2_O_2_ in methanol for 30 min. After washing three times with PBS (5 min each), tissue slices were incubated in normal blocking serum for 20 min at room temperature. After removing normal blocking serum, tissue slices were incubated with a type II collagen antibody (diluted to 1:400) and type I collagen antibody (diluted to 1:1,000) in a humidified chamber for 2 h at room temperature. After removing the primary antibody and washing with PBS three times (5 min each), slices were incubated with biotinylated secondary antibody and streptavidin-peroxidase. The activity of peroxidase was then detected using the Vector® DAB Substrate kit (3,3-diaminobenzidine; SK-4100, Vector Laboratories, CA, USA). Stained tissue slices were dehydrated using alcohol and xylene and mounted for microscopic evaluation.

### Statistical analysis

All measurements were performed in triplicate. Results are presented as the mean ± standard deviation (SD) and analyzed by *t *tests and ANOVA (IBM SPSS software, version 20.0). Differences between groups were considered statistically significant when the *p* value was less than 0.05.

## Results

### Observation mixing materials

**Figure 1:**
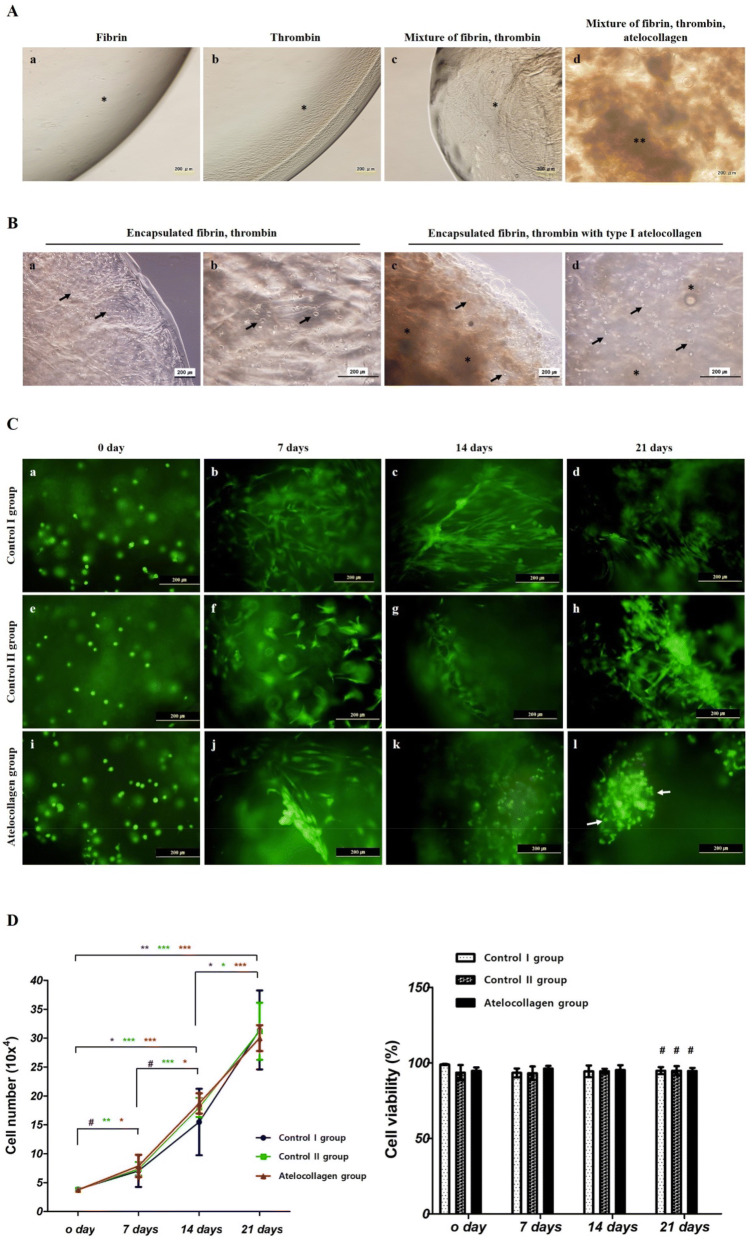
Mixture materials and cell distribution, viability and proliferation. (**A**) Observation of mixed materials. Only Fibrin (a, * indication), thrombin (b, * indication), and the mixture of fibrin and thrombin (c, * indication). The fibrin, thrombin, and atelocollagen mixtures were observed with the substances expected of collagen under a microscope (d, ** indication). Scale bar; 200 µm. (**B**) Cell and materials distribution. Gel beads made with two different mixture ratios: 2 × 10^6^ cells/0.8 mL hMSCs mixed with 0.2 mL thrombin in one syringe and 1 mL fibrin in the other syringe (a and b); and 2 × 10^6^ cells/0.8 mL hMSCs mixed with 0.2 mL thrombin in one syringe and 0.2 mL atelocollagen mixed with 0.8 mL fibrin in the other syringe (c and d). hMSCs mixed well with materials and were well distributed (a–d, black arrow). Beads maintained their round shape well after mixture bead formation. Atelocollagen was observed (c and d, * black indication). Scale bar; 200 µm. (**C**) Biochemical staining of cell viability in mixture beads. Fluorescent dye staining after mixing for control I group (mixture of fibrin, hMSCs, and thrombin cultured in basal medium), control II group (mixture of fibrin, hMSCs, and thrombin cultured in chondrogenic differentiation medium), and the atelocollagen group (mixture of fibrin, atelocollagen, hMSCs, and thrombin cultured in chondrogenic differentiation medium). Viable cells were green, and dead cells were red when they were stained with calcein acetoxymethyl ester (calcein-AM) and ethidium homodimer-1 (EthD-1). These beads showed stable cell viability in all groups during culture. Especially, as cell morphology maintained a round shape in the atelocollagen group after 21 days of culture (l, white arrow), but not in other groups. Scale bar; 200 µm. (**D**) Cell proliferation and viability. Cells counts after all group bead digestion were measured using type I collagenase on days 0, 7, 14, and 21. After day 0 and 21 of culture period, the number of cells increased from 3.837 ± 0.283 × 10^4^ to 3.142 ± 0.682 × 10^5^ in control I group, 3.784 ± 0.207 × 10^4^ to 3.12 ± 0.494 × 10^5^ in control II group and 3.758 ± 0.217 × 10^4^ to 3.002 ± 0.223 × 10^5^ in the atelocollagen group (average ± SD) (Fig. 1D, blue color; **P < 0.01, red and green color; ***P < 0.001). Cell viability of all groups was stable on day 21 with 94.8 ± 2.326% in control I group, 94.83 ± 2.926% in control II group and 94.5 ± 2.152% in the atelocollagen group (average ± SD%). There was no significant difference in the comparison of all groups (^#^*P* > 0.05).

Fibrin, thrombin, and atelocollagen without cells were observed under a microscope. Fibrin (Fig. 1A (a), *), thrombin (Fig. 1A (b), *) and mixed with fibrin, thrombin (Fig. 1A (c), *) were observed in a transparent gel. The fibrin, thrombin, and atelocollagen mixtures were observed with the substances expected of collagen under a microscope (Fig. 1A (d), **). Scale bar; 200 µm.

### Distribution of cells in mixture beads

Microscopic observations were first performed to observe the distribution of cells in the mixture of beads (Fig. 1B). Gel beads made with two different mixture ratios: 2 × 10^6^ cells/0.8 mL hMSCs mixed with 0.2 mL thrombin in one syringe and 1 mL fibrin in the other syringe (Fig. 1B (a and b); and 2 × 10^6^ cells/0.8 mL hMSCs mixed with 0.2 mL thrombin in one syringe and 0.2 mL atelocollagen mixed with 0.8 mL fibrin in the other syringe (Fig. 1B (c and d). The hMSCs were well mixed in the two different mixture ratios and maintained a round shape (Fig. 1B (a–d), black arrow). Mixed atelocollagen was observed with hMSCs encapsulated in atelocollagen beads (Fig. 1B (c and d), * black indication). Scale bar; 200 µm.

### Viability and proliferation of hMSCs in mixture beads

Viability was determined using calcein-AM and EthD-1 dyes, along with a fluorescence microscope. Fluorescent dye staining was performed after mixing for the control I group, control II group, and the atelocollagen group. Viable cells were green, and dead cells were red when they were stained with calcein acetoxymethyl ester (calcein-AM) and ethidium homodimer-1 (EthD-1). hMSC beads of all groups showed well-maintained cell survival and distribution in gel beads on day 0, 7, 14, and 21 (Fig. 1C). Images demonstrated that all hMSCs remained viable in gels and stable during the experimental period with chondrogenic differentiation in medium and basal medium conditions. In particular, hMSC beads of the atelocollagen group showed chondrocyte-like morphology in the chondrogenic differentiation medium at 21 days (Fig. 1C (l), white arrow)).

The number of cells was measured by separating the cells from the beads by type I collagenase on days 0, 7, 14, and 21. Cell numbers increased with the culture period (Fig. 1D, cell number). Cell numbers were increased with 3.784 ± 0.207 × 10^4^ cells (average ± SD) in the control II group, 3.758 ± 0.217 × 10^4^ cells in the atelocollagen group on day 0 and 7.417 ± 1.175 × 10^4^ cells in the control II group, 7.898 ± 1.916 × 10^4^ in the atelocollagen group on day 7. Cell numbers between days 0 and 7 showed a significant difference in the control II and the atelocollagen group (Fig. 1D, green and red color; *P < 0.05, **P < 0.01). The control I group did not show a significant difference on day 0 and 7 with 3.837 ± 0.283 × 10^4^ on day 0 and 7.05 ± 2.786 × 10^4^ on day 7 (Fig. 1D, blue color, ^#^*P* > 0.05). After 14 and 21 days of culture, the number of cells increased from 1.549 ± 0.575 × 10^5^ to 3.142 ± 0.682 × 10^5^ in the control I group, 1.803 ± 0.166 × 10^5^ to 3.12 ± 0.494 × 10^5^ in the control II group and 1.873 ± 0.176 × 10^5^ to 3.002 ± 0.223 × 10^5^ in the atelocollagen group. As a result, on day 0 and day 21 (the end of the incubation period), the number of cells in all groups increased, and significant results are shown (Fig. 1D, blue color; **P < 0.01, red and green color; ***P < 0.001).

Cell viability of all groups was stable on day 21 with 94.8 ± 2.326% in the control I group, 94.83 ± 2.926% in the control II group and 94.5 ± 2.152% in the atelocollagen group (average ± SD%) (Fig. 1D, cell viability). There was no significant difference in cell viability between all the groups (^#^*P* > 0.05).

### Size change of hMSC-encapsulated gel beads

**Figure 2:**
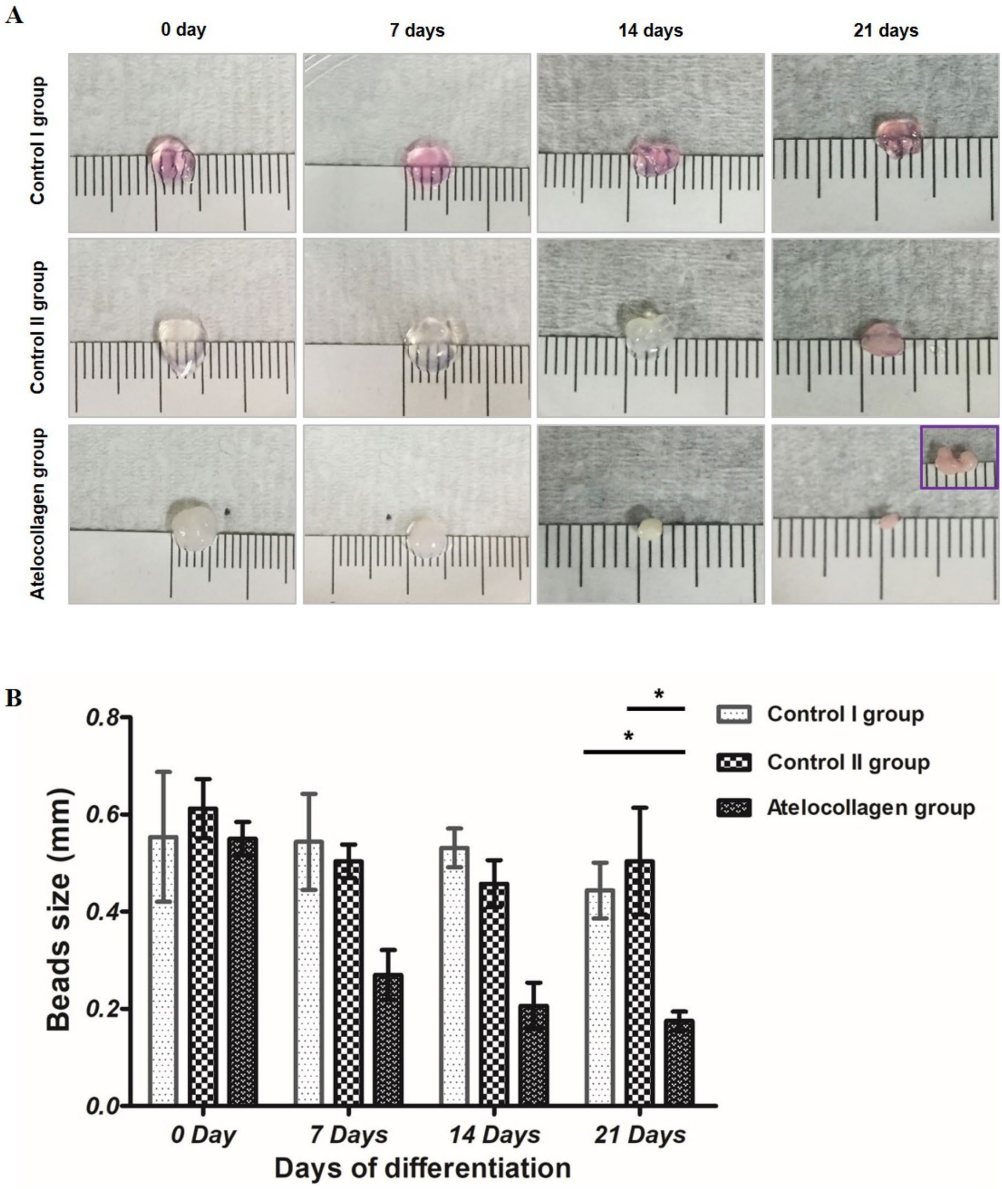
Condensation in the atelocollagen mixture beads. (**A**) Visual observation showed that beads of the control I group (mixture of fibrin, hMSCs, and thrombin cultured in basal medium), the control II group (mixture of fibrin, hMSCs, and thrombin cultured in chondrogenic differentiation medium), and the atelocollagen group (mixture of fibrin, atelocollagen, hMSCs, and thrombin cultured in chondrogenic differentiation medium) had a smooth surface. Interestingly, beads attached to form bundles in encapsulated atelocollagen (purple small box). (**B**) Measured bead diameters of the atelocollagen group were significantly decreased compared to those of other groups on day 21 (n = 8 in each group, *P < 0.001).

The sizes of hMSC beads in all groups were assessed on days 0, 7, 14, and 21. Results showed that all beads had a smooth surface (Fig. 2A). Results of bead diameter measurements on day 0, immediately after mixing, were as follows: atelocollagen group, 0.55 ± 0.035 mm; control I group, 0.554 ± 0.134 mm; control II group, 0.613 ± 0.061 mm. On day 7, measured bead diameters were as follows: atelocollagen group, 0.27 ± 0.051 mm; control I group, 0.544 ± 0.099 mm; control II group, 0.504 ± 0.034 mm. Results showed that at 7 days, beads were smaller in the atelocollagen group than in the other groups. On day 7, beads started to condense in the atelocollagen group. After 21 days of differentiation, beads in the atelocollagen group had an average diameter of 0.175 ± 0.019 mm, which was smaller than that of the control I group (0.444 ± 0.057 mm) and the control II group (0.504 ± 0.11 mm, both *P < 0.001; Fig. 2B). Interestingly, beads in the atelocollagen group were attached and formed bundles (Fig. 2A, purple box).

### Microstructures of encapsulated hMSCs in gel beads

**Figure 3:**
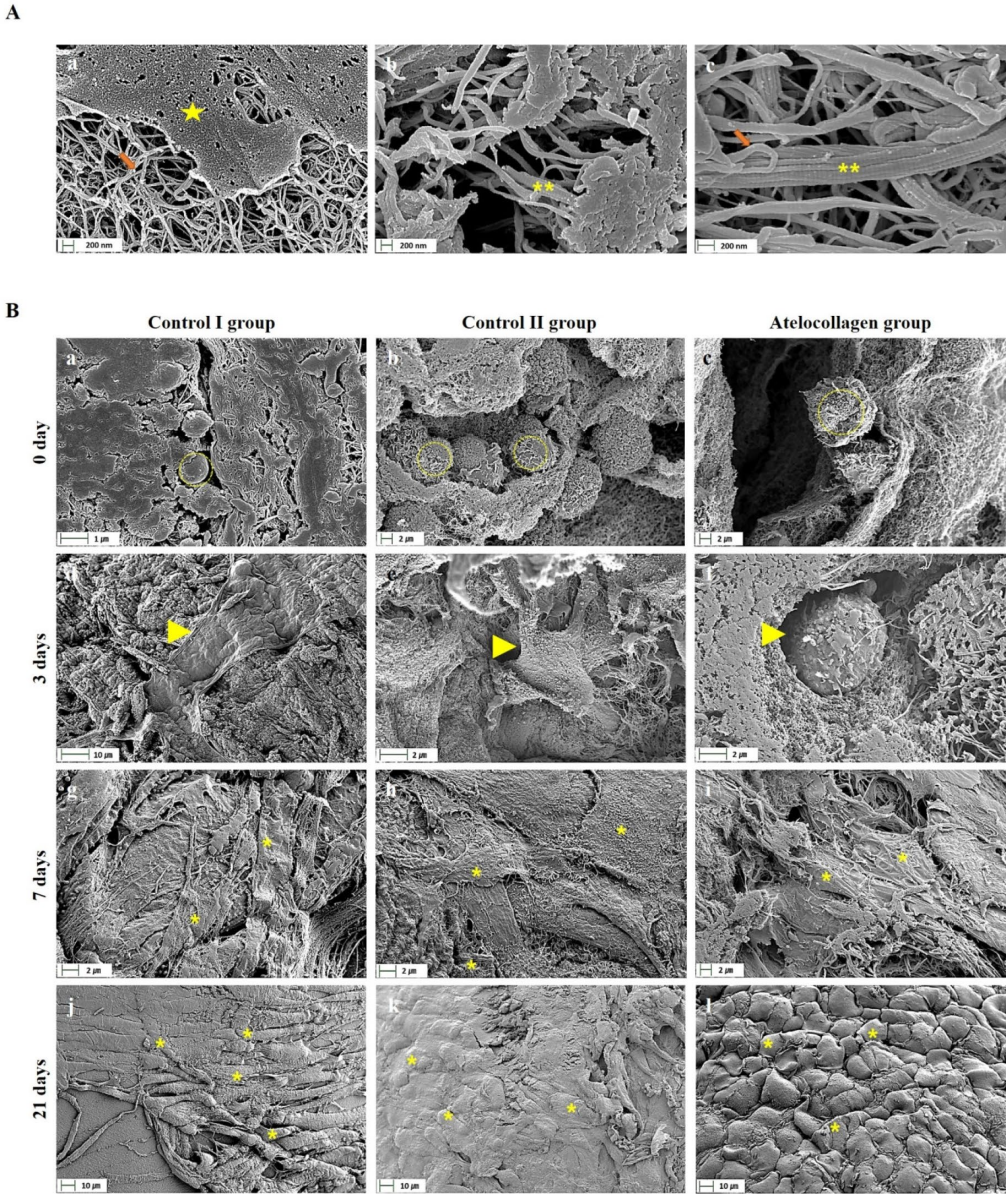
SEM image of hMSC mixture beads. (**A**) Structure in SEM of fibrin, thrombin, and atelocollagen without cells. (a) It is a mixed structure of fibrin and thrombin (fibrin; orange arrow, thrombin; yellow star). (b) The structure of only atelocollagen and the fibers are observed (**, yellow indication). (c) It is a mixed structure of fibrin, thrombin, and atelocollagen. The structures of collagen fiber (**, yellow indication) and fibrin is observed (orange arrow). (**B**) SEM image of fibrin, thrombin, and atelocollagen with cells. Control I group (mixture of fibrin, hMSCs, and thrombin cultured in basal medium; a, d, g, j), control II group (mixture of fibrin, hMSCs, and thrombin cultured in chondrogenic differentiation medium; b, e, h, k), and atelocollagen group (mixture of fibrin, atelocollagen, hMSCs, and thrombin cultured in chondrogenic differentiation medium; c, f, i, l) were investigated at 0, 3, 7, and 21 days after culture. Immediately after mixing, hMSCs showed round shape morphology in all groups (a–c, dotted line yellow circle). The atelocollagen group maintained the round shape up to 3 days after culture (f, yellow arrowhead), but the control I and II groups did not (d, e, yellow arrowhead). A large number of cells (* yellow indication) had surfaces in close contact with each other in all groups at 21 days (j–l). Chondrocyte-like morphology (spherical morphology) was only observed in the atelocollagen group (l, * yellow indication). Representative SEM images are shown. Scar bar: 200 nm (**A** (a–c)), 1 µm (**B** (a)), 2 µm (**B** (b, c, e–i)), 10 µm (**B** (d, j–l)).

In order to observe the structure of fibrin, thrombin, and atelocollagen within the gel beads, SEM was performed without cells (Fig. 3A). Within the Fibrin and thrombin mixtures without cells, fibrin was observed in the form of thin fibers (Fig. 3A (a), orange arrow) and thrombin in the form of small beads (Fig. 3A (a), yellow star). In the atelocollagen-only image, thick fiber bundles with nodes were observed (Fig. 3A (b), ** yellow star). Also, fibrin, thrombin, and atelocollagen mixed structures showed fibrous atelocollagen (Fig. 3A (c), ** yellow star), and fibrin (Fig. 3A (c), orange arrow).

**Figure 4:**
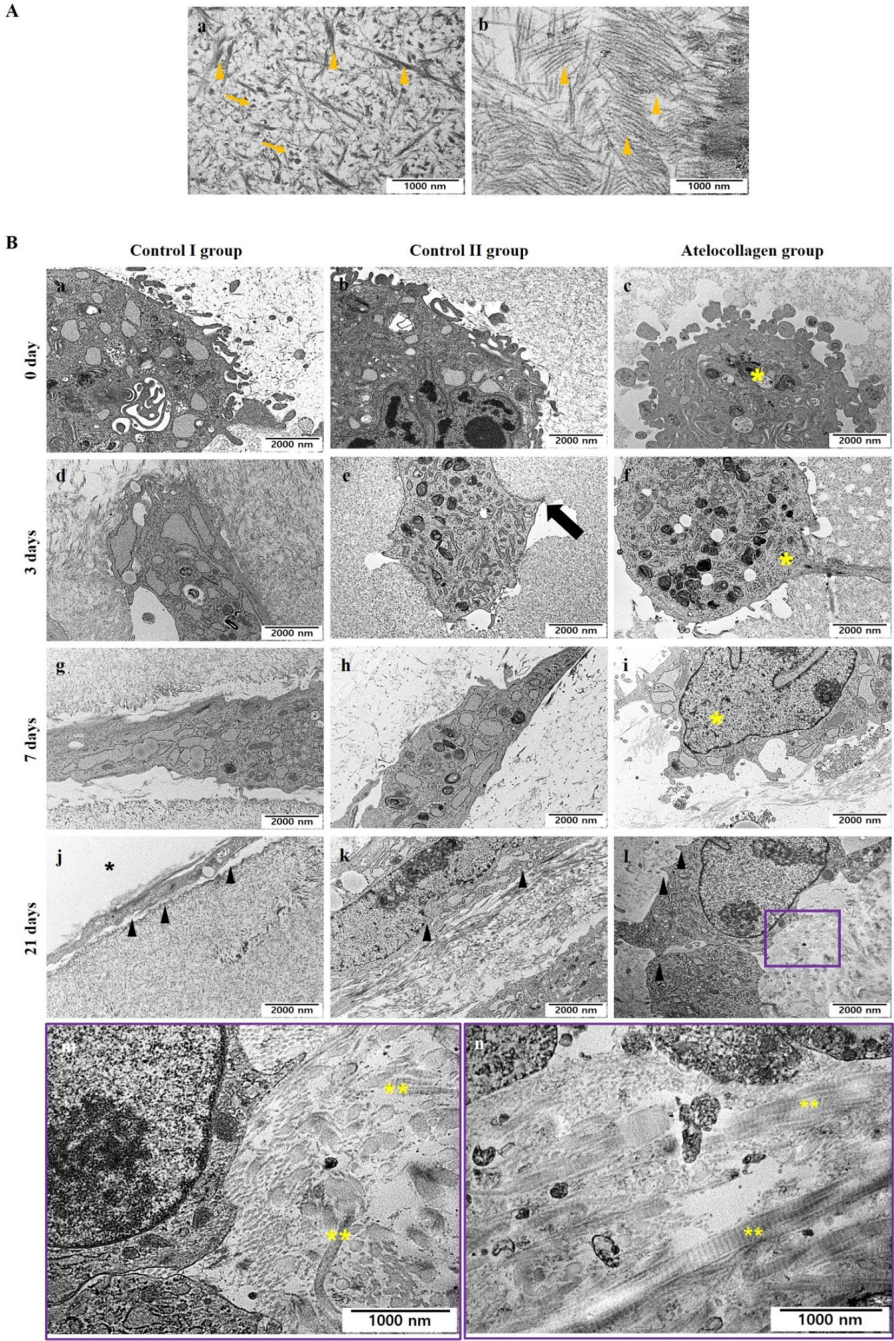
TEM of hMSC mixture beads. (**A**) Structure in TEM of fibrin, thrombin, and atelocollagen without cells. (a) It is a mixture of fibrin and thrombin. Fibrin was observed in the form of fiber (orange arrowhead) and thrombin in the form of a clot (orange arrow). (b) The structure of only atelocollagen and the fibers are observed (orange arrowhead). (**B**) TEM image of fibrin, thrombin, and atelocollagen with cells. The control I group (mixture of fibrin, hMSCs, and thrombin cultured in basal medium) (a, d, g, j), the control II group (mixture of fibrin, hMSCs, and thrombin cultured in chondrogenic differentiation medium) (b, e, h, k), and the atelocollagen group (mixture of fibrin, atelocollagen, hMSCs, and thrombin cultured in chondrogenic differentiation medium) (c, f, i, l) were investigated after days 0, 3, 7, and 21 of culture. Control I and control II groups changed from a round shape (a and b) to an elongated morphology at 3 days after culture (d and e). Cells tried to touch the matrix such as fibrin and thrombin by stretching foot-like projections (e, black arrow). However, the atelocollagen group maintained the round shape from days 0 to 7 after culture (c, f, and i, * yellow indication). The round shape remained unchanged until 21 days after culture (l), and cells extended to form contacts with the surrounding matrix (l, black arrowhead), but not other groups (j and k, black arrowhead; elongated cells). At 21 days, the matrix was enriched with collagen fiber bundles in the atelocollagen group (m and n, ** yellow indication in the purple box). The purple box of image m and n is an enlargement of the small purple box of image l. Representative TEM images are shown. Scale bar: 1,000 nm (**A** (a and b), **B** (m and n)). 2000 nm (**B** (a–l)).

The microstructures of beads of all groups were investigated by SEM and TEM after 0, 3, 7, and 21 culture days (Figs. 3 and 4). SEM images revealed that hMSCs of all the groups maintained a round shape at 0 days (Fig. 3B (a–c), dotted line yellow circle). hMSCs of control groups I and II showed a flat morphology after 3 days (Fig. 3B (d and e), yellow arrowhead). However, those in the atelocollagen group maintained a round shape similar to that observed on day 0 (Fig. 3B (f), yellow arrowhead). Cells began to wrap around the surface of the bead and touch each other after 7 days (Fig. 3B (g–i), * yellow indication).

hMSC beads of all groups showed a higher cell density on the gel surface after 21 days of culture. We observed that all cells were firmly attached without the appearance of gaps (Fig. 3B (j–l), * yellow indication). Furthermore, many fibroblast-like cells (flat and elongated morphology) accumulated on the bead surface of control groups I and II after 7 and 21 days of culture (Fig. 3B (j and k)). However, the atelocollagen group was found to comprise chondrocyte-like cells (spherical morphology) 21 days after chondrogenic differentiation (Fig. 3B (l)).

In order to observe the structure of fibrin, thrombin, and atelocollagen, which are components of the gel beads, TEM was performed without cells (Fig. 4A). In the fibrin and thrombin mixture without cells, fibrin was observed in the form of fiber (Fig. 4A (a), orange arrowhead)) and thrombin in the form of a clot (Fig. 4A (a), orange arrow). In the atelocollagen-only image, a collagen bundle with nodes was observed (Fig. 4A (b), orange arrowhead)).

TEM images showed that hMSC beads of all groups remained spherical at 0 days (Fig. 4B (a–c)). This result was similar to SEM observations. From day 3, hMSC beads of control I and II groups showed an elongated morphology (Fig. 4B (d and e)). A foot-like projection of the cells showed contact with the mixed matrix, such as fibrin and thrombin (Fig. 4B (e), black arrow). However, hMSC beads of the atelocollagen group maintained their round shape, and a portion of cells extended to form contacts with surrounding material (Fig. 4B (f), * yellow indication). In contrast, hMSC beads of control I and II groups exhibited altered cell shapes with elongated morphology 7 days after culture (Fig. 4B (g and h), whereas the beads of the atelocollagen group maintained their round shape (Fig. 4B (i), * yellow indication). On day 21, cells covered the bead surfaces (Fig. 4B (j and k), black arrowhead; elongated cell, * indication; bead outside). These results are similar to the SEM image observed. Collagen fibers were first detected in the atelocollagen group (Fig. 4B (l), purple box), and cell microvilli extended to form contacts with the surrounding matrix (Fig. 4B (l), black arrowhead). The matrix was enriched, and collagen fiber bundles were abundantly observed in hMSC beads of the atelocollagen group. These collagen bundles were thick and clearly demarcated (Fig. 4B (m and n), ** yellow indication). Image m and n is an enlarged image of the purple box.

### Gene expression

**Figure 5:**
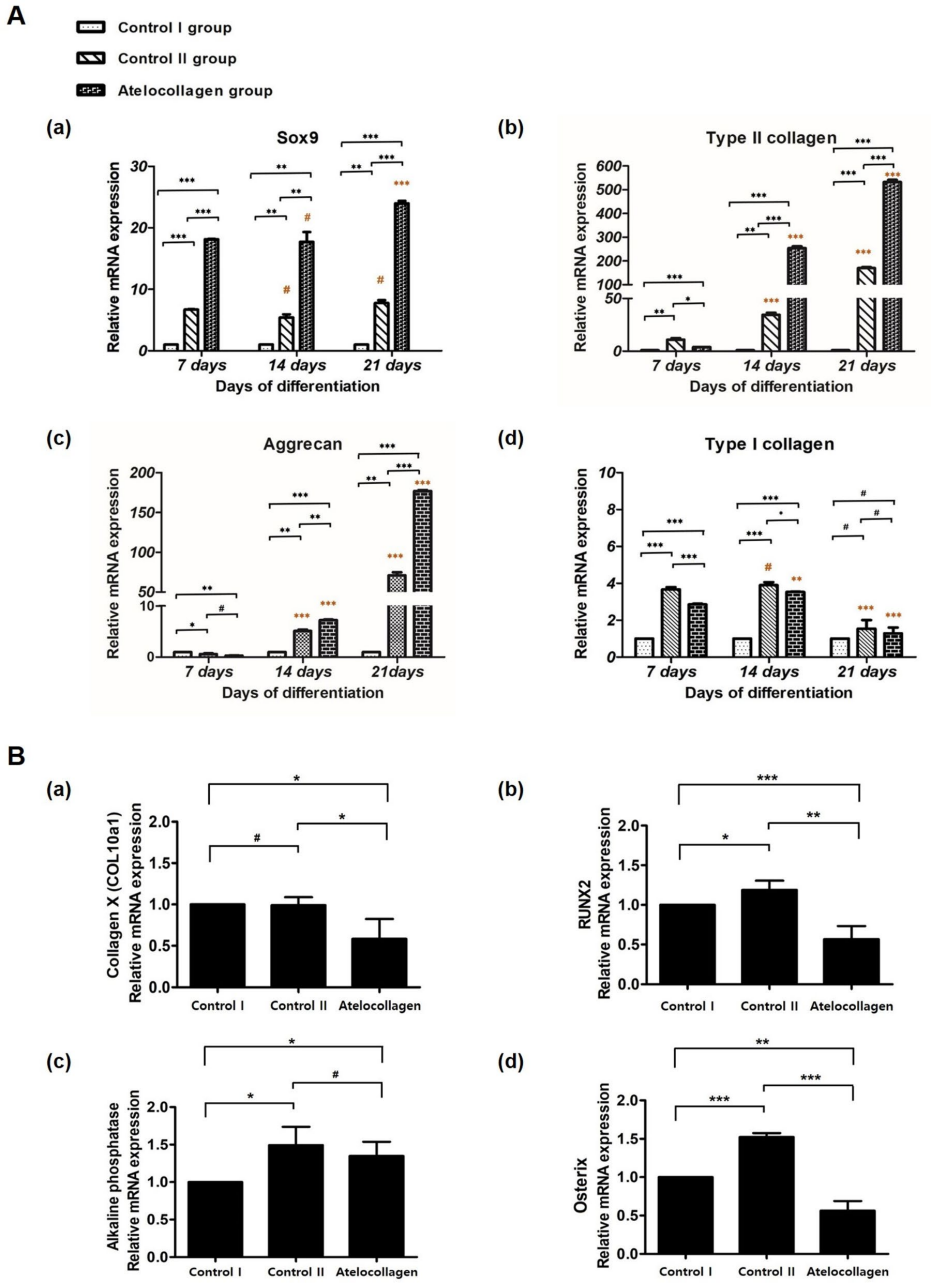
Gene expression analysis of hMSC beads. (**A**) mRNA expression levels of (a) *Sox9*, (b) *type II collagen*, (c) *aggrecan* and (d) *type I collagen* in control I group (mixture of fibrin, hMSCs, and thrombin cultured in basal medium), control II group (mixture of fibrin, hMSCs, and thrombin cultured in chondrogenic differentiation medium), and the atelocollagen group (mixture of fibrin, atelocollagen, hMSCs, and thrombin cultured in chondrogenic differentiation medium) at 7, 14 and 21 days after in vitro culture. (a) The expression level of *Sox9* in the atelocollagen group was highest 18.1-fold on day 7 to 23.9-fold on day 21 of culture and remained high during the culture period. On day 21, Sox9 expression was significantly higher compared to day 7 (***, orange indication). The expression of *Sox9* results increased early on day 7 compared to the other chondrogenic differentiation markers. (b) *Type II collagen* expression was highly upregulated 254.3-fold on day 14 and 532.6-fold on day 21 of culture in the atelocollagen group. Among the other chondrogenic differentiation markers, the *type II collagen* expression value was the highest on day 21 in the atelocollagen group. Expression values on day 21 were significant compared to day 7 (***, orange indication). (c) The *aggrecan* expression of the atelocollagen group increased rapidly 0.3-fold on day 7 to 176.9-fold on day 21, and a significant value was shown (***, orange indication). (d) Nevertheless, *type I collagen* expression was increased 3.7- to 3.9-fold in the control II group and 2.9- to 3.5-fold in the atelocollagen group on days 7 and 14. However, on day 21, *type I collagen* expression decreased 1.5-fold in the control II group and 1.3-fold in the atelocollagen group. Decreased values on day 21 were significant when compared with day 7 (***, orange indication). * Black indication: Compare expression values of groups on the same day. * Orange indication: Comparison of expression values by day 7 (early stage). (**B**) To evaluate the mRNA expression levels of (a) Type X *Collagen (COL10A1)*, (b) *Runx2,* (c) *ALP,* and (d) *Osterix* in the control I, control II and atelocollagen groups on day 21 after in vitro culture. (a) *CoL10A1* expression was decreased 0.58-fold on day 21, which was significant in the atelocollagen group compared to the control I group (*). (b) *Runx2* expression was slightly increased by 1.19-fold in the control II group. The atelocollagen group expression was significantly decreased by 0.57-fold in the atelocollagen group compared to the control I group (***). Expression in the control II group was significantly increased by 1.52-fold in (d) *Osterix* (***) and 1.49-fold in (c) *ALP* marker (*) compared to the control I group. Expression in the atelocollagen group was decreased by 0.56-fold in *Osterix* (**) and 1.35-fold in *ALP* marker (*). n = 3 in each group. ^#^*P* > *0.05,* *P < 0.05, **P < 0.01, ***P < 0.001.

To evaluate the differentiation of hMSC beads, RT-qPCR analyses were performed to determine gene expression levels of three key chondrogenic differentiation markers, namely *Sox9, aggrecan, type II collagen*, and the highly expressed fibrocartilage marker *type I collagen*, in bead mixture constructs on day 7, 14, and 21 of culture (Fig. 5A). Also, on day 21 of differentiation culture, the ossification markers *Runx2,* ALP, type X collagen *(COL10A1),* and *Osterix* expression were observed (Fig. 5B).

The *Sox9* transcription factor is a marker initially expressed in early chondrogenic differentiation^46^. *Sox9* expression showed an early 6.7-fold upregulation in the control II group and 18.1-fold in the atelocollagen group on day 7. The expression of other differentiation markers, such as type II collagen and aggrecan, was delayed (Fig. 5A (a)). In the control II group, the expression of *Sox9* increased by 5.4-fold as compared to the control I group on day 14 and 7.8-fold on day 21. However, the increase between day 14 and day 21 was not statistically significant. The expression of *Sox9* significantly increased from 17.7-fold to 23.9-fold in the atelocollagen group on days 14–21. The expression level of *Sox9* in the atelocollagen group was the highest during the culture period. The expression of *Sox9* on day 21 was significantly increased compared to the early stage (day 7) of chondrogenic differentiation (Fig. 5A (a), orange indication, ^#^*P* > 0.05, ***P < 0.001). Also, when comparing the control I group with another group at the same time point, there was a significant difference in the *Sox9* expression values of the control II and atelocollagen groups (Fig. 5A (a), black indication, **P < 0.01, ***P < 0.001).

Aggrecan expression increased between 5.1- and 70.9-fold in the control II group and 7.2- to 176.9-fold in the atelocollagen group from days 14 to 21 (Fig. 5A (c)). The aggrecan expression of the atelocollagen group increased rapidly, with the highest values observed on day 21. The increased aggrecan expression was significant in the control II group and atelocollagen groups on days 14 and 21 when compared with day 7 of early chondrogenic differentiation (Fig. 5A (c), orange indication, ***P < 0.001). When comparing the control I group with other groups at the same time point, there was a significant increase in the aggrecan expression values of the control II and atelocollagen groups on days 14 and 21 (Fig. 5A (c), black indication, ^#^*P* > 0.05, *P < 0.05, **P < 0.01, ***P < 0.001).

In the atelocollagen group, the type II collagen expression was highly upregulated by as much as 3.9-fold by day 7, 254.3-fold by day 14, and 532.6-fold by day 21 of culture (Fig. 5A (b)). In the control II group, type II collagen expression increased 10.92-fold by day 7, 34.48-fold by day 14, and 169.5-fold by day 21. Among the three groups, the atelocollagen group had the highest value on day 21, and significant results were shown compared with day 7 (Fig. 5A (b), orange indication, ***P < 0.001). Also, when comparing the control I group with another group at the same time point, expression of type II collagen was significant in the control II and atelocollagen groups on day 14 and 21 (Fig. 5A (b), black indication, **P < 0.01, ***P < 0.001).

Type I collagen expression was increased 3.7- to 3.9-fold in the control II group and 2.9- to 3.5-fold in the atelocollagen group compared with the control I group on days 7 and 14 (Fig. 5A (d), black indication, *P < 0.05, ***P < 0.001). However, on day 21, type I collagen expression decreased to a 1.5-fold difference in the control II and 1.3-fold in the atelocollagen group compared with the control I group. The increased type I collagen expression in control II and atelocollagen groups on day 21 were not statistically significant relative to the control I group (Fig. 5A (d), black indication, ^#^*P* > 0.05). Additionally, type I collagen expression at day 21 was significantly decreased in control II and atelocollagen groups when compared to day 7 of early chondrogenic differentiation (Fig. 5A (d), orange indication, ***P < 0.001).

The *type X collagen* gene (*COL10A1)* is specifically expressed during hypertrophy of maturation chondrocytes as an endochondral ossification process^43^. *Runx2*, *ALP*, and *Osterix* work together in hypertrophic chondrocyte-specific *CoL10A1* gene expression^44^. *CoL10A1* expression was decreased 0.58-fold on day 21 and represented a significant difference in the atelocollagen group compared to the control I group (Fig. 5B (a), *P < 0.05). *Runx2* expression was increased slightly 1.19-fold in the control II group (Fig. 5B (b), *P < 0.05). However, the atelocollagen group expression was significantly decreased 0.57-fold compared to the control I group (Fig. 5B (b), ***P < 0.001). *Osterix* expression was similar to the *Runx2* expression pattern (Fig. 5B (d)). *Osterix* expression of the control II group was significantly increased by 1.52-fold (***P < 0.001), but the expression in the atelocollagen group was 0.56-fold decreased (**P < 0.01) compared to the control I group. Unlike other ossification markers, ALP expression was increased 1.49-fold in the control II group (Fig. 5B (c) *P < 0.05) and 1.35-fold in the atelocollagen group (*P < 0.05) compared to the control I group.

### Examination of chondrogenic differentiation by ELISA

On day 21 of chondrogenic differentiation, the supernatant of the culture medium was harvested after centrifugation to collect all proteins. ELISA was performed to detect levels of aggrecan and type II collagen (*COL2A1*) as cartilage markers; type I collagen as a fibrocartilage marker; and MMP13 as an ossification marker in the supernatants of all groups.

**Figure 6:**
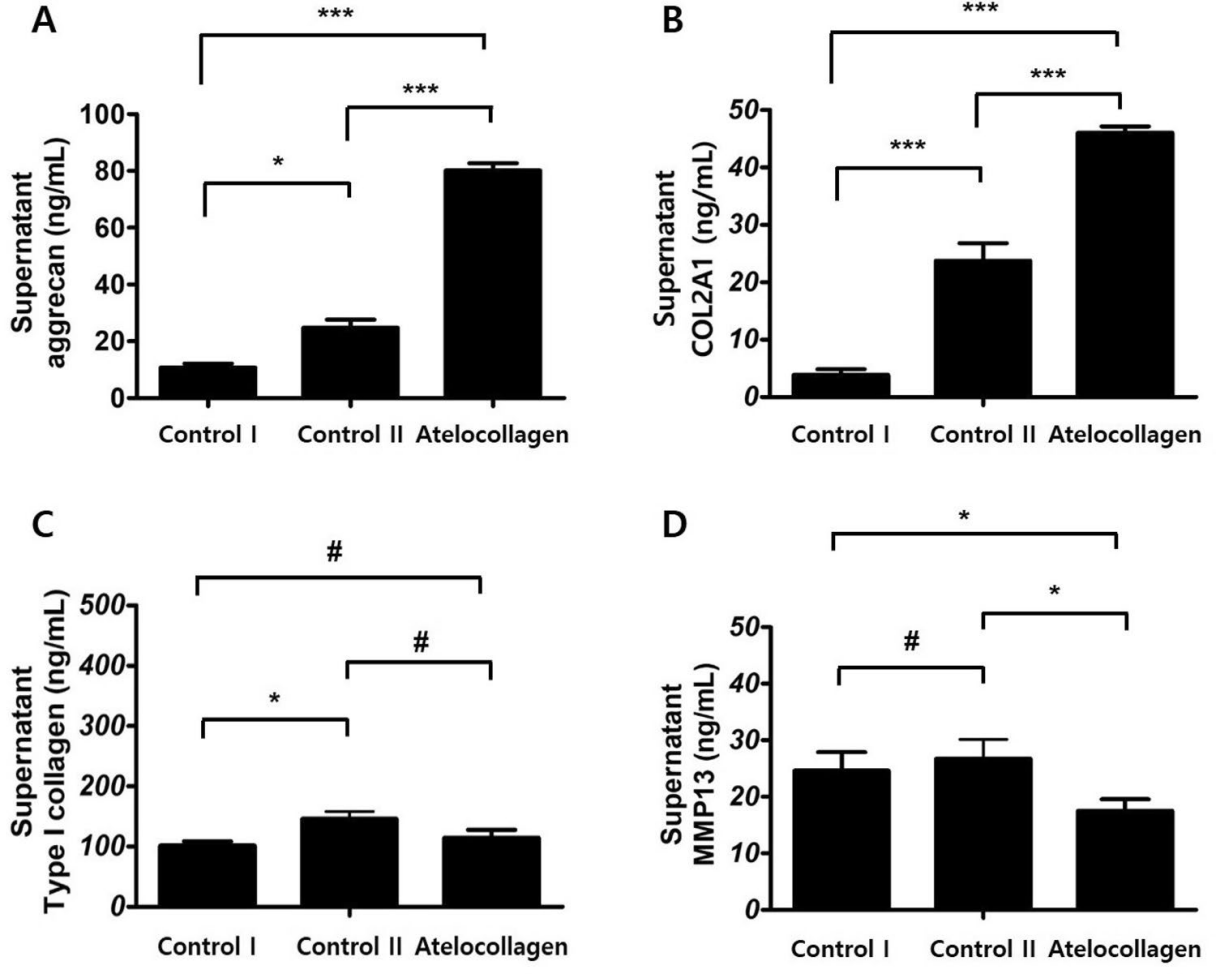
Expression of cartilage matrix-related protein in hMSC beads. Protein expression levels in the culture supernatants from control I group (mixture of fibrin, hMSCs, and thrombin cultured in basal medium), control II group (mixture of fibrin, hMSCs, and thrombin cultured in chondrogenic differentiation medium), and atelocollagen group (mixture of fibrin, atelocollagen, hMSCs, and thrombin cultured in chondrogenic differentiation medium) were analyzed by ELISA. (**A**) Aggrecan levels in the culture media of the atelocollagen group showed higher expression as compared to both the control I and II groups (***). (**B**) COL2A1 expression showed similar trends to aggrecan expression (***). (**C**) Type I collagen expression was slightly increased in the control II group (*), however, the expression of the atelocollagen group was not (^#^). (**D**) MMP13 was only slightly expressed in the atelocollagen group. The results were significant compared to the control I group (*). n = 3 in each group. ^#^*P* > 0.05, *P < 0.05, **P < 0.01, ***P < 0.001.

Aggrecan expression was highest in the atelocollagen group among all the groups. Results were significant compared to the control I and control II groups (Fig. 6A, *P < 0.05, ***P < 0.001). The COL2A1 expression value in the atelocollagen group was the highest when compared to other groups and demonstrated similar trends to those observed with aggrecan expression. Significant results are shown in Fig. 6B (***P < 0.001). On the other hand, type I collagen expression was slightly increased (Fig. 6C, *P < 0.05) in control II group. The atelocollagen group expression value was not significant (^#^*P* > 0.05) compared to the control I or control II groups (Fig. 6C). MMP13 value was lowly expressed in the atelocollagen group (Fig. 6D). This expression was significant compared to the control I group (*P < 0.05).

### Histological examination

**Figure 7:**
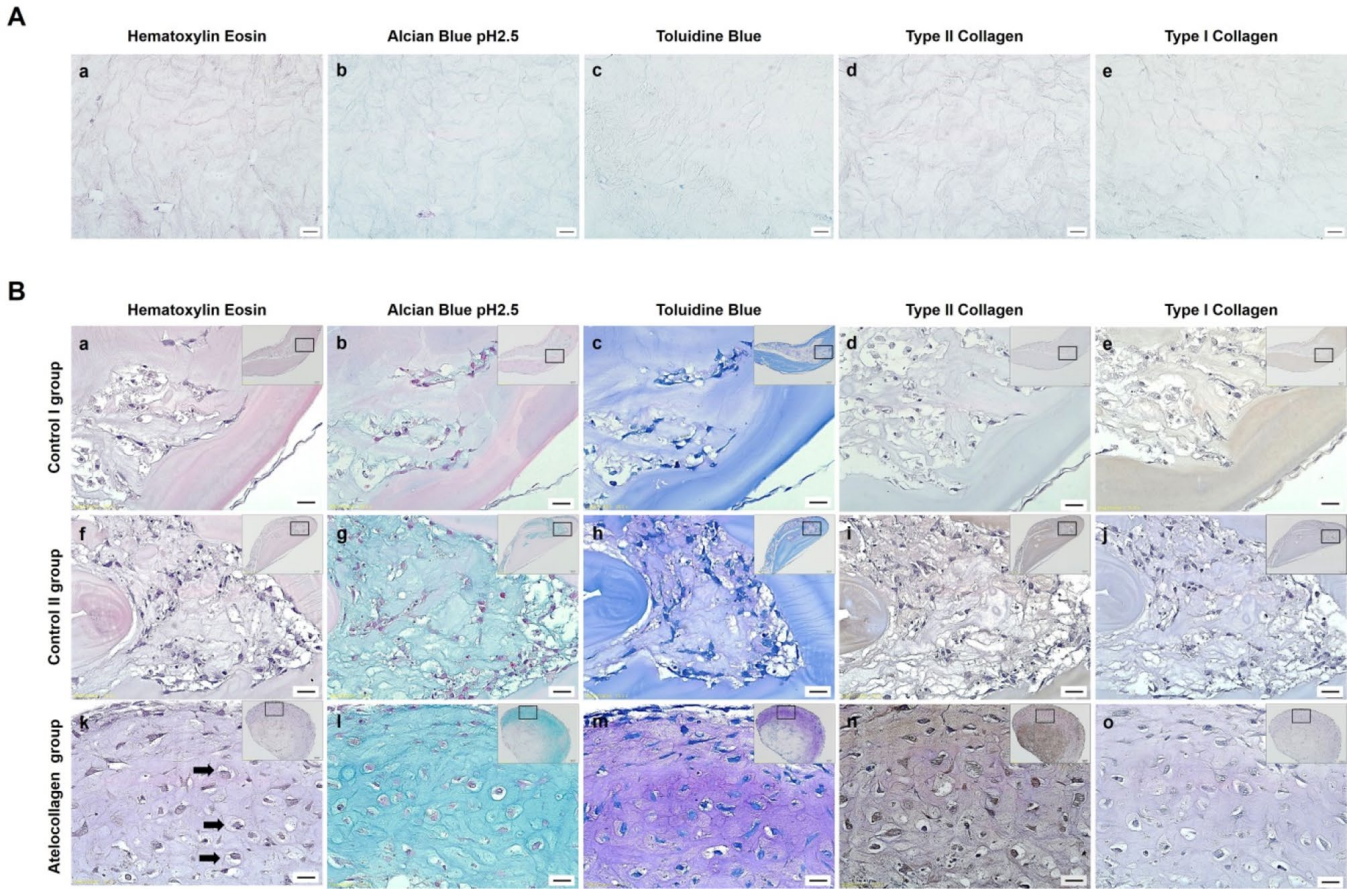
Histological analysis of chondrogenic markers in hMSC beads. (**A**) Staining of fibrin, thrombin, and atelocollagen in the absence of cells. The gel beads without cells were cultured in chondrogenic differentiation medium on day 21. (a) Hematoxylin–eosin demonstrated weak eosin staining (weak pink color) and one large mass without empty space. (b) Weak Alcian blue pH 2.5 and (c) toluidine blue staining was observed. (e) Type I and (d) type II collagen staining did not demonstrate brown expression. Scale bar: 20 µm. (**B**) Staining of fibrin, thrombin, and atelocollagen with cells. Representative histological images of the control I group (mixture of fibrin, hMSCs, and thrombin cultured in basal medium), control II group (mixture of fibrin, hMSCs, and thrombin cultured in chondrogenic differentiation medium), and atelocollagen group (mixture of fibrin, atelocollagen, hMSCs, and thrombin cultured in chondrogenic differentiation medium) at 21 days after culture. Hematoxylin–eosin staining showed well mixed cells and a structure similar to lacunae (k, black arrow) compared to the other groups (a and f). Alcian blue pH 2.5 and toluidine blue staining showed expression around cells in the atelocollagen group (l and m), and the control I and II groups contained weak expression in the densely packed cells (b and g, c and h). Type II collagen was highly expressed around chondrocyte-like cells in the atelocollagen group (n) and weakly expressed in the control II group (i) compared to the control I group (d). Type I collagen staining was not observed in the control II and atelocollagen groups (j and o). Nevertheless, gel beads of the control I group were weakly expressed (e). Scale bar; 20 µm.

For histological analysis, hematoxylin and eosin staining were performed to evaluate changes in cell morphology. Alcian blue, toluidine blue, type II collagen of cartilage markers, and type I collagen of fibrocartilage marker staining was performed to assess chondrogenic expression patterns in all groups after 21 days of culture. The gel beads of fibrin, thrombin, and atelocollagen without cells were observed compared to the gel beads of fibrin, thrombin, and atelocollagen with cells (Fig. 7A).

The gel beads of fibrin, thrombin, and atelocollagen without cells showed as one mass without empty space (Fig. 7A). Following hematoxylin and eosin staining, gel beads without cells showed weak eosin staining (weak pink color) (Fig. 7A (a)). Alcian blue PH 2.5 and toluidine blue staining expressed weakly (Fig. 7A (b and c)). Immunohistochemistry staining for type I collagen and type II collagen staining was not observed with brown expression of DAB (Fig. 7A (d and e)).

The gel beads of fibrin, thrombin, and atelocollagen with cells were observed (Fig. 7B). Hematoxylin and eosin staining showed fibrin, thrombin, and atelocollagen well mixed with cells (Fig. 7B (a, f and k)). The cells of the atelocollagen group showed large round nuclei compared to other groups at 21 days of culture. We also observed a structure similar to lacunae in the cartilage tissue of the atelocollagen group (Fig. 7B (k), black arrow), but not in other groups (Fig. 7B (a and f)). Alcian blue pH 2.5 and toluidine blue staining demonstrated the accumulation of GAGs. Its expression was observed around rounded cells, and the structure was similar to lacunae in the atelocollagen group (Fig. 7B (l and m)). GAGs expression in the control I and control II groups were weakly expressed in the densely packed areas of the cells (Fig. 7B (b and g, c and h)). Immunohistochemistry staining for type II collagen and type I collagen was performed for all groups 21 days after culture. Immunohistochemistry for type II collagen demonstrated the presence of cartilage-specific ECM. Intracellular type II collagen as a matrix component was brown expressed around chondrocyte-like cells in the atelocollagen group (Fig. 7B (n)) and weakly expressed in the control II group (Fig. 7B (i)) compared to the control I group (Fig. 7B (d)). Type I collagen staining was not observed in the control II and atelocollagen groups (Fig. 7B (j and o)). However, gel beads of the control I group were weakly expressed (Fig. 7B (e)).

## Discussion

In this study, we evaluated differences in hMSC chondrogenic potential according to diverse compositions of fibrin, thrombin, and atelocollagen. These compositions are commonly used clinically for cartilage regeneration. Fibrin and thrombin are used for cell delivery to cartilage defects, and atelocollagen is used to enhance chondrogenic differentiation. However, basic research on these compositions has rarely been reported.

Cell survival, differentiation, marker expression, histologic analysis, and immunohistochemical examinations were performed to determine the chondrogenic differentiation of hMSC beads among all groups. Our main findings in this study were as follows: (1) atelocollagen enhanced the chondrogenic potential of MSCs based on Alcian blue pH 2.5 staining, toluidine blue staining, collagen type II immunohistochemistry, and RNA expression analysis (*collagen II*, *aggrecan*, and *Sox9*) after 21 days of chondrogenic differentiation; (2) atelocollagen scaffolds provided improved conditions for chondrogenic differentiation without affecting cell viability, based on electron microscopy findings.

The clinically optimal composition of 2 × 10^6^ hMSCs/0.2 mL mixed with 0.2 mL thrombin in one syringe and 0.2 mL atelocollagen mixed with 0.8 mL fibrin in the other syringe was used in this study based on a previous paper^47^. Many scaffold types, such as sponges, sheets, and gels, have been reported to be suitable for cartilage regeneration^48–51^. The three-dimensional structure of the scaffold can facilitate cell proliferation, differentiation, and colonization. Fibrin and thrombin, used in this study, are helpful for the formation of three-dimensional structures for cartilage regeneration^52–56^. Fibrin was previously found to improve chondrocyte survival and activity both in vitro^57^ and in vivo^58,59^. However, this is not sufficient for cartilage regeneration as cartilage has limited repair potential. Besides, it has not been successfully used for clinical treatment. New and better materials with enhanced chondrogenic potential for cartilage regeneration are thus needed.

The atelocollagen used in this study was produced from pig skin through a salt precipitation method. This method has marginal detrimental effects on natural collagen structure while preserving biocompatibility. Regarding the immunogenicity of collagen, atelocollagen is safe as it has been depleted of telopeptide, which can cause immune reactions; specifically, telopeptides are removed from tropocollagen through a pepsin treatment procedure.

Although the major collagen component of articular cartilage is type II collagen, type I collagen scaffolds are being used clinically. Type I collagen is a component of the cartilage ECM with aggrecan and GAGs. When type I collagen scaffolds are used with MSCs, a signal for chondrogenic differentiation can be induced. Furthermore, these scaffolds can recruit host MSCs in vivo, which can differentiate into chondrocyte-like cells^41,50,60^. In addition, microstructures of gel-type atelocollagen are similar to those of the articular cartilage ECM, and they can stimulate MSCs to actively produce ECM^61–63^. When chondrocytes are cultured together with type I collagen, chondrocyte morphology is retained, and GAGs are well expressed in vitro^64,65^. Our study showed that type I atelocollagen provided improved conditions for chondrogenic differentiation based on scaffold biocompatibility, as it had no immunogenic component. The weak histological expression of type I collagen in the atelocollagen group may be due to atelocollagen telopeptide depletion(Fig. 7A.e). Additionally, the type 1 expression can become diminished by several manufacturing processes, including dissolution and neutralization of the pH. Type I collagen gene expression in the atelocollagen group was observed in the early culturing stage, suggesting that atelocollagen is an excellent cell stimulator for initial matrix production, resulting in the production of type I collagen. However, as time progressed, within the continuous chondrogenic environment, the cells began to differentiate into the chondrogenic lineage, with subsequent reduction in the amount of type I collagen produced during the 21 days of culture.

Articular cartilage is hyaline cartilage, and the most crucial matrix component is type II collagen. Type VI collagen is present in the pericellular region, whereas type X collagen is found in the calcified layer^66^. Type I collagen is a component of the matrix and is highly expressed in fibrocartilage^67^. Therefore, it is difficult to detect type I collagen in mature hyaline cartilage. During chondrogenic differentiation, MSCs first encounter the fibrocartilage-like matrix, such as type I collagen fibers. As shown in this study, isolated MSCs can interact with the mixed atelocollagen and undergo condensation and differentiation, and these processes are necessary for chondrogenic differentiation. Type I collagen fibers are known to provide differentiation signals to the cell surface. Other studies have also used type I collagen scaffolds to induce MSC condensation and chondrogenic differentiation^68,69^. However, our study showed definitive evidence of these processes. Here, cell condensation and chondrogenesis were observed in hMSC beads of the atelocollagen group after 21 days of culture. Results also showed a reduction in bead size after 7 days of culture, implying the occurrence of condensation. Furthermore, on day 21, beads in the atelocollagen group were the smallest among all groups (Fig. 2).

Chondrogenic differentiation was also analyzed by determining levels of cartilage-specific markers such as Sox9, type II collagen, and aggrecan^70^. Among these cartilage-specific markers, Sox9 is a transcription factor that plays a vital role in controlling type II collagen and aggrecan expression during chondrogenic differentiation. Its expression occurs early in chondrogenic differentiation^46^, and it plays an essential role in cell condensation and differentiation^71,72^. In this study, hMSCs encapsulated in atelocollagen expressed Sox9 after 7 days of culture. As the differentiation progressed (on day 21), aggrecan and type II collagen expression was observed as chondrogenic markers (Fig. 6). In addition, bead sizes decreased in a time-dependent manner in the atelocollagen group, which may correlate with the chondrogenesis condensation process. Accordingly, hMSC condensation and Sox9 expression increased with chondrogenic differentiation^46,71,72^. As a result, a chondrocyte-like appearance with lacunae was observed in cartilage tissue and phenotype^73^ with the expression of glycosaminoglycans (GAGs) observed via Alcian blue pH 2.5 and toluidine blue staining (Fig. 7).

Endochondral ossification is an important event that is essential to mammalian skeletal development. Endochondral ossification begins with the condensation of multipotent MSCs, the formation of the chondrogenic matrix, and differentiation into chondrocytes^74,75^. These chondrocytes become more differentiated into hypertrophic chondrocytes^76^. At this time, cartilage tissue begins to be replaced by bone tissue, through apoptosis of chondrocytes, cartilage matrix degradation, and vascular invasion was generated^76,77^. Hypertrophic differentiation of chondrocytes is the main problem with MSCs for cartilage repair. It is crucial to inhibit hypertrophic chondrocytes in MSC application in cartilage tissue engineering^78^. We observed the expression of chondrogenic differentiation markers, including Sox9, aggrecan, and type II collagen (COL2A1) in the atelocollagen group. In contrast, Runx2, Osterix, and MMP13 expression reduced in the atelocollagen group. Runx2 and Osterix play important roles in cartilage hypertrophy^75^. Also, these are induced in MMP13 and type X collagen (COL10A1) expression during chondrocyte differentiation. Interestingly, many studies suggest that Sox9 inhibits the maturation of chondrocytes^79–81^. We consider that the expression of Sox9 in the atelocollagen group inhibited the hypertrophy of the chondrocytes and helped maintain the expression of cartilage differentiation marker at the end of differentiation (on day 21) and decreased the expression of ossification markers.

In this study, type I collagen mRNA expression was observed at 7–14 days of early stage chondrogenic differentiation in the atelocollagen group. Type I collagen is expressed at the end of fibrocartilage differentiation^82,83^, but its expression during chondrogenic differentiation is known to provide the signal for cell condensation and differentiation^84^. Therefore, during hMSC chondrogenic differentiation, type I collagen is abundantly expressed in the ECM in the early stages. The expression of type I collagen also stimulates chondrogenic cell condensation, morphological changes in cells, and further chondrogenic differentiation process^69^. During this process, integrin on the cell surface and type I collagen interact, focal adhesion kinase (FAK) is activated, and the cell adhesion factors such as N-Cadherin and N-CAM increase^85,86^. As a result, cells can attract each other, the spacing between cells becomes narrow, and the possibility of cell–cell contact increases. This process leads to intracellular signaling and induces the expression of Sox9, which is a key factor in the induction of chondrogenic differentiation^68,69,86^.

Type I atelocollagen used in this study, mixed with hMSCs, could provide a three-dimensional structure for cell survival and chondrogenic differentiation. Type I atelocollagen is abundantly present in ECM together with other components during the chondrogenic differentiation process. Furthermore, it can act as a signal for hMSC condensation to enhance differentiation.

In conclusion, hMSCs are good cell sources that can be easily harvested with chondrogenic differentiation potential for cartilage tissue engineering. Our results show that type I atelocollagen can enhance the chondrogenic differentiation of hMSCs and demonstrate that type I atelocollagen is a suitable support for in vitro cartilage tissue engineering applications.

## Supplementary information

Supplementary information

Supplementary Video 1

## Data Availability

All data generated or analyzed during this study are included in this published article (and its Supplementary Information files).
