# Supplementary material for: Atelocollagen promotes chondrogenic differentiation of human adipose-derived mesenchymal stem cells
Source: Sci Rep. 2020 Jun 30;10:10678. doi: 10.1038/s41598-020-67836-3 (PMC7327030; doi:10.1038/s41598-020-67836-3)
Supplement: Supplementary file 1 — Supplementary information [file 41598_2020_67836_MOESM1_ESM.pdf]

Atelocollagen promotes chondrogenic differentiation of human adipose-derived mesenchymal stem cells.

Seon Ae Kim[a], Yoo Joon Sur[a], Mi-La Cho[b], Eun Jeong Go[a], Yun Hwan Kim[a], Asode Ananthram Shetty[c], Seok Jung Kim [a], *

[a]Department of Orthopedic Surgery, College of Medicine, The Catholic University of Korea, Seoul, Republic of Korea

[b]The Rheumatism Research Center, Catholic Research Institute of Medical Science, College of Medicine, The Catholic University of Korea, Seoul, Republic of Korea

[c]The Institute of Medical Sciences, Faculty of Health and Wellbeing, Canterbury Christ Church University, UK

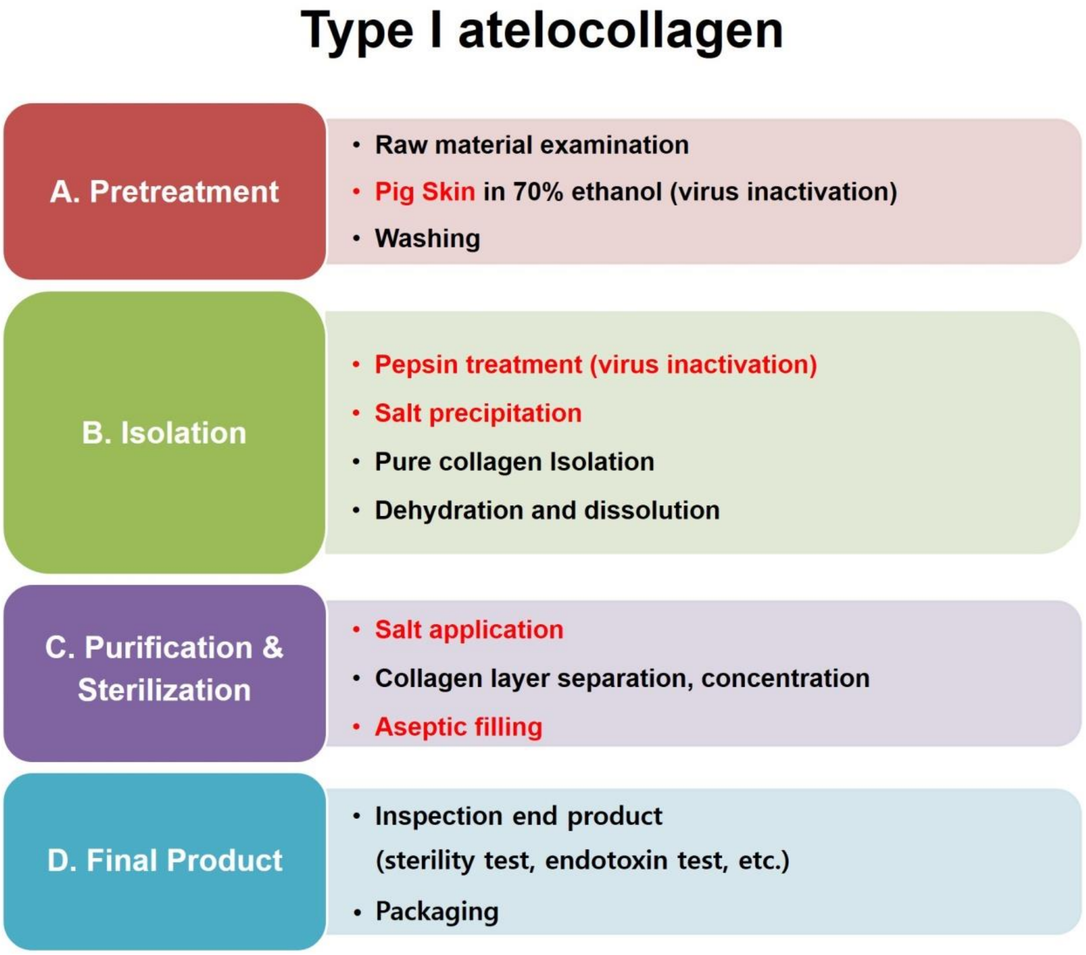

Supplementary Figure 1. Isolation of atelocollagen from pig skin

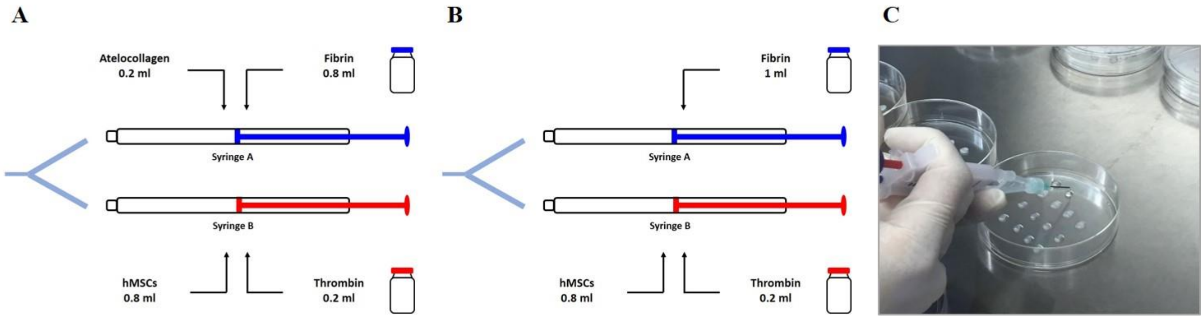

Supplementary Figure 2. Two different mixture ratios of gel beads.

(A) 2 × 10^6 hMSCs/0.8 mL, mixed with 0.2 mL thrombin in one syringe and 0.2 mL atelocollagen mixed with 0.8 mL fibrin in the other syringe. (B) 2 × 10^6 hMSCs/0.8 mL mixed with 0.2 mL thrombin in one syringe and with 1 mL fibrin in the other syringe. (C) Dropping and gel formation of hMSC gel mixture.

Supplementary Video 1. Dropping of hMSCs and gel mixture.
